# Adaptive control of a soft pneumatic actuator using experimental characterization data

**DOI:** 10.3389/frobt.2023.1056118

**Published:** 2023-03-15

**Authors:** Yoeko Xavier Mak, Hamid Naghibi, Yuanxiang Lin, Momen Abayazid

**Affiliations:** Robotics and Mechatronics Group, Faculty of Electrical Engineering, Mathematics and Computer Science, Technical Medical (TechMed) Centre, University of Twente, Enschede, Netherlands

**Keywords:** data-driven control (DDC), pneumatic actuator, minimally invasive surgery (MIS), adaptive control, fiber reinforced actuators, experimental characterisation

## Abstract

Fiber reinforced soft pneumatic actuators are hard to control due to their non-linear behavior and non-uniformity introduced by the fabrication process. Model-based controllers generally have difficulty compensating non-uniform and non-linear material behaviors, whereas model-free approaches are harder to interpret and tune intuitively. In this study, we present the design, fabrication, characterization, and control of a fiber reinforced soft pneumatic module with an outer diameter size of 12 mm. Specifically, we utilized the characterization data to adaptively control the soft pneumatic actuator. From the measured characterization data, we fitted mapping functions between the actuator input pressures and the actuator space angles. These maps were used to construct the feedforward control signal and tune the feedback controller adaptively depending on the actuator bending configuration. The performance of the proposed control approach is experimentally validated by comparing the measured 2D tip orientation against the reference trajectory. The adaptive controller was able to successfully follow the prescribed trajectory with a mean absolute error of 0.68° for the magnitude of the bending angle and 3.5° for the bending phase around the axial direction. The data-driven control method introduced in this paper may offer a solution to intuitively tune and control soft pneumatic actuators, compensating for their non-uniform and non-linear behavior.

## 1 Introduction

The compliant nature of soft pneumatic actuator has led to its widespread development for minimally invasive surgery (MIS) applications, as shown in the review paper by Runciman et al. (2019). However, these soft mechanisms have lower overall precision and repeatability compared to rigid link actuators (Burgner-Kahrs et al., 2015; Katzschmann et al., 2015). Moreover, fiber reinforcement is often added to soft actuators for controlling the mode of deformation and improve the power transfer. On the other hand, fiber reinforcement increases the non-linear response, the complexity of modeling, and control of such actuators (Fras and Althoefer, 2019; Sun et al., 2021).

Previous studies on fiber reinforced soft pneumatic actuator (SPA) design use reinforcements inside the pneumatic module, such as works by Li et al. (2020) and Dawood et al. (2021), or on the exterior of the soft module, such as works by Wirekoh et al. (2020) and Singh et al. (2018). Although internal reinforcement designs are modular and the internal deformation is more uniform, external reinforcement designs are more space efficient, which is needed for MIS.

There are two main control methods for soft actuators based on the usage of the model by the controller: model-based and data-driven controllers (George Thuruthel et al., 2018).

Modeling of soft mechanisms is more difficult and computationally intensive, compared to rigid body systems, because of its complex shape deformation. Various real time modeling and control approaches for a soft pneumatic actuator based on analytical modeling have been demonstrated, such as using Cosserat rod mechanics (Renda et al., 2018; Chikhaoui et al., 2019; Till et al., 2019), beam theory (Ataka et al., 2020), and Langrangian formulation (Sadati et al., 2018). An alternative approach to analytical modeling for control is to use the output of a finite element (FE) model in the controller framework (Faure et al., 2012; Duriez, 2013). FE model can also be used to control a soft robot adaptively through the use of gain-scheduling controllers (Wu and Zheng, 2021).

Data-driven or black-box controllers do not require explicit input of parameters of the physical system during development and tuning. For this reason, these approaches perform better for systems that are highly influenced by non-linearities and hard-to-model ‘real-world effects’ such as friction (Vikas et al., 2016), hysteresis caused by the soft material or actuation behaviour (Truby et al., 2020), and non-uniformity of the soft robot design (Giorelli et al., 2015). An example of a data-driven control method has been presented by Bruder et al. (2021), using a Koopman system identification in combination with a model predictive control (MPC) framework. An approach using supervised learning to develop a black-box model for control, tested on a cable-driven soft robot, has been introduced by Bern et al. (2020). An alternative data-driven control can be achieved using reinforcement learning (RL). Ansari et al. (2018) introduced a hybrid tendon and pneumatically actuated soft module that was controlled by an RL agent, optimizing its end-effector position and module stiffness. Despite the advantage in accuracy for these cases, data-driven controllers generally are harder to tune intuitively compared to model-based approaches, since the system behavior is driven by the learned/identified input-output mappings.

In this study, we present the design, characterization, and control of a fiber reinforced soft pneumatic actuator for the application of endoscopic surgery. We developed a data-driven control framework to compensate the ‘real-world effects’ such as non-linear mechanics and non-uniformity of the physical module while maintaining the ability to tune the controller behavior intuitively through the adaptive controller framework. Specifically, our contributions are: 1) introduction of a 12 mm diameter fiber reinforced SPA for endoscopy application, 2) mechanical characterization of the soft pneumatic actuator for its bending and force performance, 3) control of a soft pneumatic actuator using an adaptive controller based on the experimentally obtained characterization curve.

## 2 Soft pneumatic actuator module

In this section, we present the design, finite element modeling, and fabrication method of the fiber reinforced SPA employed in this study.

### 2.1 Design rationale

The design of the actuator module is based on the use case for endoscopic intervention, where the developed module will be fitted to the tip of the endoscope. This work focuses on the development of a single-module soft actuator and its control method. In particular, tool designs such as the addition of endoscopic working channels, a camera, and illumination are not included in our current study.

For endoscopic surgery, the soft actuator is required to have 2 degrees of freedom (DOF) bending motion in order to have the same capability as currently used endoscopes for gastro-intestinal or colon interventions. This already brings an improvement to the currently used endoscopes, by ensuring compliance at the material level, the soft actuator reduces the risk of tissue damage and punctures. The design choices and requirements of the SPA are presented in Table 1.

**TABLE 1:** Design choices and requirements of the fiber reinforced soft pneumatic endoscope module.

Requirement	Technical parameter	Motivation
Compliance	Young’s modulus between 10 kPa and 1,000 MPa (Rus and Tolley, 2015)	distributed & soft actuation mechanism
Size	12 mm outer diameter size	comparable to existing commercial endoscopes, max. 13.2 mm for colon & 12.8 mm for gastroendoscopy (PENTAX Medical, 2021)
Force	mean retraction force ≥3.53 N for straight and ≥0.5 N for retroflexed configuration	required forces for endoscopic biopsy procedure (Jamidar et al., 2008)

To achieve the required 2 DOF bending motion, we designed the actuator to have 3 pneumatic actuation chambers within the module. Although 2 DOF bending can theoretically be performed only using 2 separate actuation chambers, we opted to have a symmetrical arrangement using 3 chambers. Actuation using only two chambers would require the module to be prebent, or actuated using negative pressure (vacuuming), which makes the actuator response asymmetric between left-right (and up-down).

**FIGURE 1:**
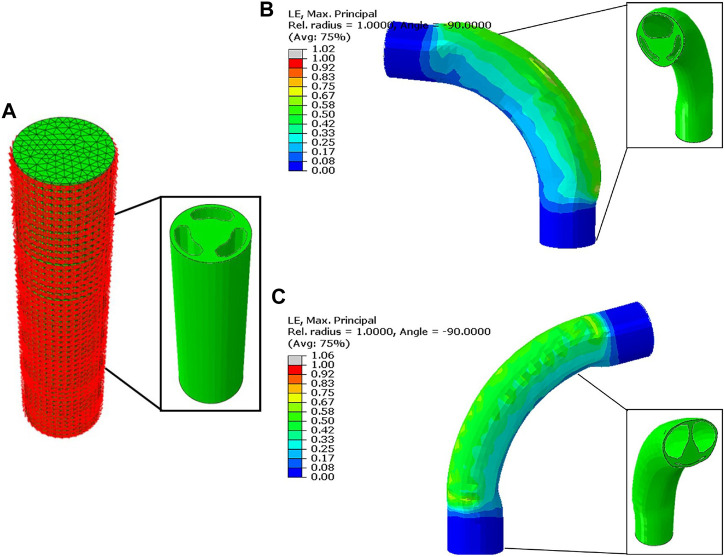
The developed FE model of the soft pneumatic actuator with the outer fiber reinforcement constraint **(A)**. The bending performance is simulated for the case of single chamber pressurization **(B)**, and pressurization of two chambers at once **(C)**.

We designed the geometry of the SPA, such that the cross-sectional area of the pneumatic actuation chambers is maximized, while allowing extra space in the center for further development of the endoscope (e.g., cables for the camera, endoscope working channels, etc.). This will improve the actuation force as the force is the cross-section area size times the chamber pressure difference to the ambient pressure. This resulted in an arc-shaped chamber design as seen in Figure 1A. Fiber reinforcement is added to the exterior of the soft module to limit excessive radial deformation.

### 2.2 Finite element modeling

To speed up the design process of the SPA, a finite element (FE) model was developed using Abaqus v2018 (Simulia, United States). The developed FE model helps to estimate the mechanical behavior of the proposed actuator without going through the lengthy fabrication process repeatedly. The CAD model of the module was imported and meshed with 10-node modified quadratic tetrahedron (C3D10M) elements. A Neo-Hookean hyper-elastic isotropic model was used to model the mechanical behavior of the inner module, in which the strain energy function *ψ* is described as a function of the first invariant of the left Cauchy-Green deformation tensor *I*_1_ and the elastic volume ratio *J*:

$$\psi_{\text{module}}=C_{10}\left(I_{1}-3\right)+\frac{1}{2D}{\left(J-1\right)}^{2},$$ (1)

where *C*_10_ and *D* are the Neo-Hookean constants and the inverse of the bulk modulus, respectively. Holzapfel-Gesser-Ogden hyperelastic material model was utilized to model the behavior of the outer fiber reinforcement. The strain energy function is described as a function of Neo-Hookean terms, representing the non-collagenous matrix, and *I*_4(*αα*)_, pseudo-invariants of $\bar{C}$ and *A*_*α*_ (directions of the fibers in the reference configuration):

$$\psi_{\text{fiber}}=C_{10}\left(I_{1}-3\right)+\frac{1}{2D}\left(\frac{J^{2}-1}{2}-\text{ln}J\right)+\frac{k_{1}}{2k_{2}}\left(e^{k_{2}{\left\langle E_{\alpha }\right\rangle }^{2}}-1\right)$$ (2)

with:

$$E_{\alpha }=\kappa \left(I_{1}-3\right)+\left(1-3\kappa \right)\left(I_{4\left(\alpha \alpha \right)}-1\right)\;$$ (3)

The constants *k*_1_ and *k*_2_ are material parameters and *κ* describes the level of dispersion in the fiber directions. Based on our previous study (Lenssen et al., 2019), the material coefficients for the module and the fiber reinforcement were adjusted from the material data sheet and bending experiments (*C*_10_ = 0.1 MPa, *D* = 0.01 MPa^−1^, *k*_1_ = 70 MPa, *k*_2_ = 0.3, *κ* = 0.1). Pressure was applied with a linear profile over time and (quasi-static) implicit analysis was completed using a standard Abaqus solver.

### 2.3 Soft pneumatic module fabrication

**FIGURE 2:**
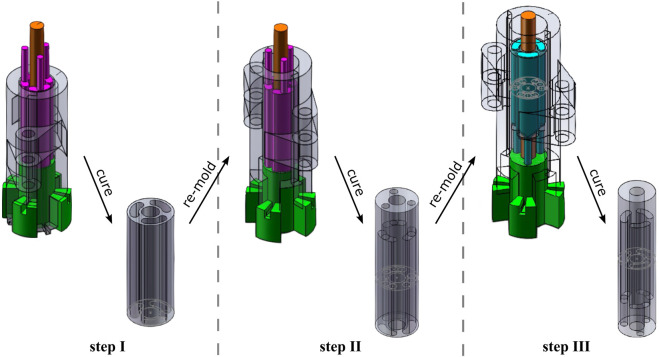
The module casting process is divided into three steps: the section with air chambers is molded in step I, while the foot and cap section is fabricated in step II and III respectively. For all steps, the silicone resin is poured from the top side of the mold.

The inner module of the soft pneumatic actuator was made of silicone material (EcoFlex 0050, Smooth-On Inc.). Before the casting process, the silicone resin was vacuumed to remove air bubbles from the material. Subsequently, the resin was poured into a custom-made modular mold in a three-step casting process, as shown in Figure 2. We used a braided polyester cable sleeve as the external fiber reinforcement of the actuator. The cable sleeve is compressed to the same length as the inner silicone module (Supplementary Material S1), and then each end of the cable sleeve is glued to the inner module.

### 2.4 Setup

**FIGURE 3:**
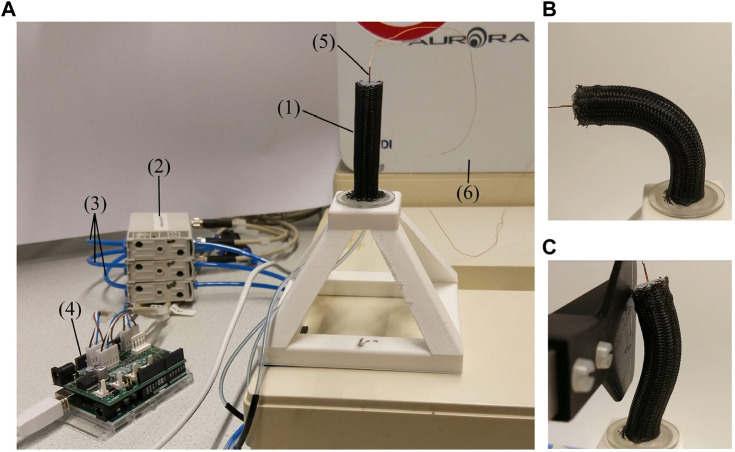
The experimental setup **(A)** for characterization and control of the soft pneumatic endoscope, consisting of: (1) the soft pneumatic module, (2) three pneumatic regulators connected to (3) pressurized air input (2 bar), (4) an Arduino with driver board, and (5) an electromagnetic pose sensor with (6) the EM-field generator. Figures **(B)** and **(C)** show the soft pneumatic module during motion and force characterization, respectively.

Figure 3 shows the setup used to characterize and control the fabricated soft pneumatic module. The air pressures of the chambers were modulated using three pressure regulators (VEAB-L-26-D2-Q4-V1-1R1, Festo AG & Co. KG, Germany). The regulators were connected to an Arduino Uno (Arduino, United States), augmented with a driver board to power and send control signals to the regulators. To measure the orientation of the soft module end effector, a miniature 6 DOF electromagnetic (EM) NDI Aurora sensor (Northern Digital, Canada) was placed at the tip of the module. Additionally, a calibrated load cell was fixed in place and was used to measure the bending force in the radial direction, as shown in Figure 3C.

## 3 Modeling

The module can be controlled in 3 DOF using 3 actuation chambers, e.g., *θ*, *ϕ*, *ℓ* which respectively are the end-effector bearing angle in the lateral direction, the phase angle of the bending plane around the vertical axis, and the length of the module. However, the elongation movement has practically limited use for endoscopic applications, as the insertion movement of the overall endoscope tool covers a larger change of length compared to the extension limit of the actuator. Therefore, we opted to control only the bending angles, actuating a maximum of 2 chambers simultaneously, to maximize the bending angle *θ*, which occurs when *ℓ* is minimum.

### 3.1 Robot kinematics

Piecewise constant curvature (PCC) kinematic assumption is used, enabling rigid-body techniques to be used for flexible mechanisms (Webster and Jones, 2010). The forward kinematics of the proposed SPA is constructed using this PCC assumption, as presented for the case of generic continuum robots by Webster and Jones (2010). However in our case, we used the bearing angles in each of the chamber’s bending plane as the actuator space variables *q*_*i*_, instead using the chamber’s lengths.

The transformation from the base of the module to end-effector is given by:

$$T_{\text{PCC}}=\left[\begin{array}{cc} I_{3\times 3} & p_{\text{foot}}\\ 0 & 1 \end{array}\right]T_{\text{CC}}\left[\begin{array}{cc} I_{3\times 3} & p_{\text{cap}}\\ 0 & 1 \end{array}\right],$$ (4)

where $p_{\text{foot}}={\left[\;0\;0\;\ell_{\text{foot}}\;\right]}^{\text{T}}$ , $p_{\text{cap}}={\left[\;0\;0\;\ell_{\text{cap}}\;\right]}^{\text{T}}$ . *T*_PCC_ is the piecewise constant curvature forward kinematics, given as the combination of bending the soft module around the *y*-axis, and then rotating the entire module around the *z*-axis.

$$T_{\text{CC}}=\left[\begin{array}{cc} R_{z}\left(\phi \right) & 0\\ 0 & 1 \end{array}\right]\left[\begin{array}{cc} R_{y}\left(\theta \right) & p_{\text{CC}}\\ 0 & 1 \end{array}\right].$$ (5)

where ***p***_CC_ is the translation vector between the start to the end of the constant curvature section, given by $p_{\text{CC}}={\left[\;r\left(1-\text{cos}\theta \right)\;0\;r\text{sin}\theta \;\right]}^{\text{T}}$ and *r* = *ℓ*_body_/*θ*.

Following Eq. 5, the general constant curvature forward kinematics is written as:

$$T_{\text{PCC}}=\left[\begin{array}{cccc} \text{cos}\phi \text{cos}\theta & -\text{sin}\phi & \text{cos}\phi \text{sin}\theta & p_{x}\\ \text{sin}\phi \text{cos}\theta & \text{cos}\phi & \text{sin}\phi \text{sin}\theta & p_{y}\\ -\text{sin}\theta & 0 & \text{cos}\theta & p_{z}\\ 0 & 0 & 0 & 1 \end{array}\right],$$ (6)

where

$$\left[\begin{array}{c} p_{x}\\ p_{y}\\ p_{z} \end{array}\right]=\left[\begin{array}{c} \text{cos}\phi \left(\ell_{\text{cap}}\text{sin}\theta -\ell_{\text{body}}\left(\text{cos}\theta -1\right)/\theta \right)\\ \text{sin}\phi \left(\ell_{\text{cap}}\text{sin}\theta -\ell_{\text{body}}\left(\text{cos}\theta -1\right)/\theta \right)\\ \ell_{\text{foot}}+\ell_{\text{cap}}\text{cos}\theta +\ell_{\text{body}}\text{sin}\theta /\theta \end{array}\right].$$ (7)

**FIGURE 4:**
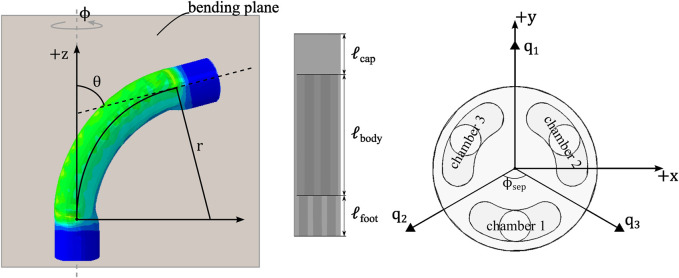
Kinematic representation of the PCC segment for the soft pneumatic endoscope module.

This forward kinematics is extended to the actuator space (*q*_*i*_ where *i* = 1, 2, 3), which is the end-effector orientation projected on the plane of bending of each respective chamber. These actuator space variables describe the bending contribution of each chamber towards the overall orientation of the actuator module. While *θ* and *ϕ* describe the orientation of the end-effector, where *θ* is the angle of deviation from the straight orientation, and *ϕ* is the phase angle around the vertical axis, as shown in Figure 4.

The configuration space variables *θ* and *ϕ* are expressed in *q*_*i*_ as:

$$\begin{array}{cc} \theta & =\sqrt{q_{1}^{2}+q_{2}^{2}+q_{3}^{2}+2\left(q_{1}q_{2}+q_{2}q_{3}+q_{1}q_{3}\right)\text{cos}\phi_{\text{sep}}},\\ \phi & =\text{atan}2\left(q_{1}+\left(q_{2}+q_{3}\right)\text{cos}\phi_{\text{sep}},\left(q_{3}-q_{2}\right)\text{sin}\phi_{\text{sep}}\right), \end{array}$$ (8)

where *ϕ*_sep_ is the separation angle between each bending plane. The value of *ϕ*_sep_ = 132° is obtained experimentally during the characterization process. This value is higher than 120° due to the expansion of the chambers during actuation, as shown in Figure 1C.

### 3.2 Motion characterization

The general dynamics model of a generic soft robot (Della Santina et al., 2020) can be written as:

$$M\left(q\right)\ddot{q}+\left(C\left(q,\dot{q}\right)+D\right)\dot{q}+G\left(q\right)+Kq=u,$$ (9)

where *M*(*q*) is the robot’s inertia matrix, $C\left(q,\dot{q}\right)$ is the Coriolis and centrifugal terms, *D* is the damping term, *G*(*q*) is the gravity effect on the robot, and *K* is the stiffness matrix. For a soft robot made of hyperelastic material, the stiffness changes according to the strain of the material; therefore, the stiffness also depends on the bending angle, that is, *K*(*q*). Since the robot currently does not carry a payload (camera or other tools) and the actuator is light in terms of mass (4.4 g), the contribution of *G*(*q*) is small with respect to the other terms.

The identification of *K*(*q*) was performed using characterization experiments, in which the module is actuated slowly to not excite the dynamic terms. When only a single chamber is actuated at a time, the bending angle at the end-effector, *q*_*i*_ ∈ *q* is mapped to the chamber’s input pressure *u*_*i*_ through:

$$k_{i}\left(q_{i}\right)q_{i}=u_{i}$$ (10)

Combining Eqs 8 and 10, the static map from input pressures to the orientation of the end effector is obtained.

## 4 Experimental characterization

Based on the model described in the previous section, we characterized the relationship between the angle of bending *θ* and the pressure of the chamber *u*_*i*_. Furthermore, a force characterization experiment was conducted to evaluate the amount of lateral force the designed SPA can exert.

### 4.1 Steady-state motion characterization

In the motion characterization experiment, the chambers were actuated one at a time. In this case, the end-effector bending angle *θ* is equal to the angle of the actuation chamber *q*_*i*_, following Eq. 8. Therefore, the mapping between end-effector angle *θ* and input pressure *u*_*i*_ during this characterization process is described in Eq. 10.

Characterization was performed using ascending and descending pressure sweeps to account for hysteresis behavior (4 cycles for each chamber). The tip orientation had to reach steady state before the next input pressure value was sent to the regulator, to prevent dynamic effects. The step change in the pressure values was set to the resolution of the pressure regulator (Δ*u*_*i*_ = 0.00195 bar). All actuator chambers are preloaded with 0.05 bar nominal pressure to avoid the discontinuous behavior of the pressure regulator during the initialization phase and to fill the remaining gap between the inner silicone module and the outer fiber, so that the module can immediately bend when pressure is applied.

Similar procedures were performed for two-chamber actuation at the same time, *u*_*i*_ = *u*_*j*_ and (*i*, *j*) = (1, 2) (2, 3), (1, 3). These two-chamber actuation experiments are performed to validate if the single-chamber characterization curve can be used to estimate bending in other directions. The maps obtained from the two-chamber actuation experiments are not used for the control of the actuator. The actuator angles *q*_*i*_ with *i* = 1, 2, 3, obtained in the single-chamber characterization, are substituted in Eq. 8 to calculate the estimated bending angle $\hat{\theta }\left(u_{i},u_{j}\right)$ in the direction of the two-chamber activation. The estimated angle $\hat{\theta }\left(u_{i},u_{j}\right)$ is then compared with the experimentally measured *θ*(*u*_*i*_, *u*_*j*_), to assess whether the single-chamber characterization results can be extended to the entire robot’s workspace in 2D. This is important for constructing the feedforward and feedback control over the whole workspace.

We expected hysteresis in the measurement of the characterization curve, which is caused by the intrinsic behavior of the elastomer and the friction between the outer reinforcement fiber and the inner silicone module. To quantify hysteresis in the characterization curve, the percentage difference between the measured bending angle during the ascending phase (*q*_asc_) and the descending phase (*q*_desc_) is given as

$$h_{q}=\frac{\left|q_{\text{asc}}-q_{\text{desc}}\right|}{\text{max}\left(q_{\text{asc}}\right)}\cdot 100\%$$ (11)

The hysteresis percentage *h*_*q*_ was calculated for all single chamber characterization curves.

**FIGURE 5:**
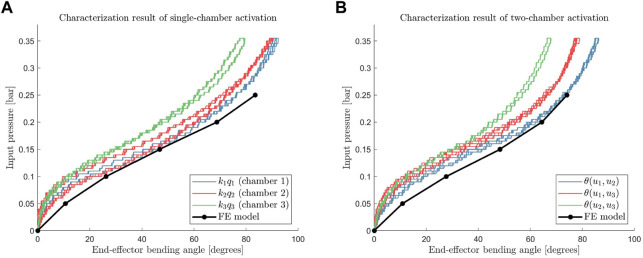
Angle to input pressure relation at steady-state when only single actuation chamber is pressurized **(A)**, and two actuation chambers are pressurized simultaneously **(B)** along with each finite element model outputs.

The results of the characterization experiments are presented in Figure 5. The input pressure and the bending angle are presented on the vertical and horizontal axes, respectively, to mimic the hyperelastic stress-strain curve at the material level. Furthermore, the result of the characterization experiment is compared with the FE simulation results. The characterization curve follows a shape similar to that of the FE simulation output. The variation between results for the three bending planes (for each chamber) can be attributed to non-uniformity of the prototype due to the manual casting process. Furthermore, uneven compression of the outer reinforcement fiber layer during the fabrication process might affect the maximum bending for the different bending planes. Although the module behaves non-uniformly, the motion is repeatable. Figure 5 shows that the resulting curve from the four ascending and descending cycles coincides for all three cases.

The means of hysteresis *h*_*q*_ are 3.82%, 6.70%, and 2.63%, for the characterization of chamber 1, chamber 2, and chamber 3, respectively. These values are comparable to the amount of hysteresis found in the EcoFlex 00-50 tensile test (Liao et al., 2020), which is used as the soft material of the inner module.

**FIGURE 6:**
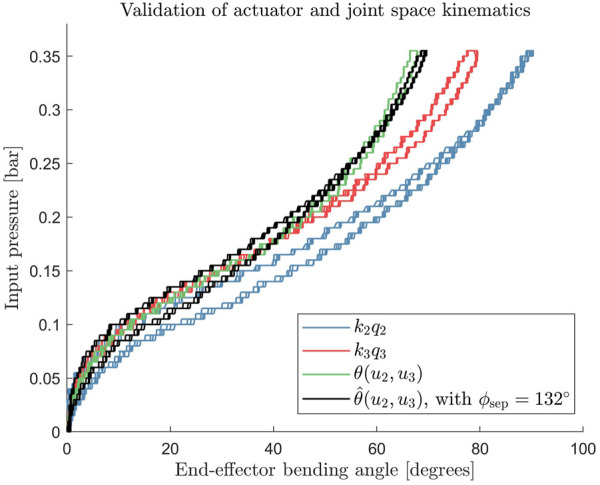
Comparison between estimated curve using the generalized 2D kinematics (shown in black) to the experimentally measured bending data (green) shows agreement for the simultaneous activation of chamber 2 and 3.

Furthermore, we compare the results of two-chamber activation experiments with an estimate constructed using the single-chamber characterization data. The experimentally measured curves of two-chamber activations are compared with the calculated estimate $\hat{\theta }\left(u_{i},u_{j}\right)$ for various chamber combinations, to validate whether the obtained characterization curve *K*_*i*_ *q*_*i*_ can be generalized to the 2D workspace using the kinematics model. As an example, the estimated bending angle $\hat{\theta }\left(u_{2},u_{3}\right)$ and the measured bending angle *θ*(*u*_2_, *u*_3_) are shown for the combination of chambers 2 and 3. The estimate coincides with the experimentally measured values, as shown in Figure 6, except for a small deviation when the input pressures are close to the maximum operating pressures. This agreement was also found for other chamber combinations (*i*, *j*) = (2, 3) and (1,3). Based on these validations, Eq. 10 can be extended to the entire workspace:

$$K\left(q\right)q=u.$$ (12)

The characterization curve can be fitted and utilized to construct a control input signal that compensates for the stiffness term in the model.

### 4.2 Force characterization

In addition to the previous setup, a strain gauge load sensor connected to a rigid force plate was used to measure this interaction force (Figure 3). The load sensor was calibrated using a set of calibrated masses prior to the force characterization experiment. In this case, the force was measured only in the lateral direction since the axial movement is not controlled. Consequently, in our current implementation, the axial force depends only on the inherent stiffness of the actuator.

**FIGURE 7:**
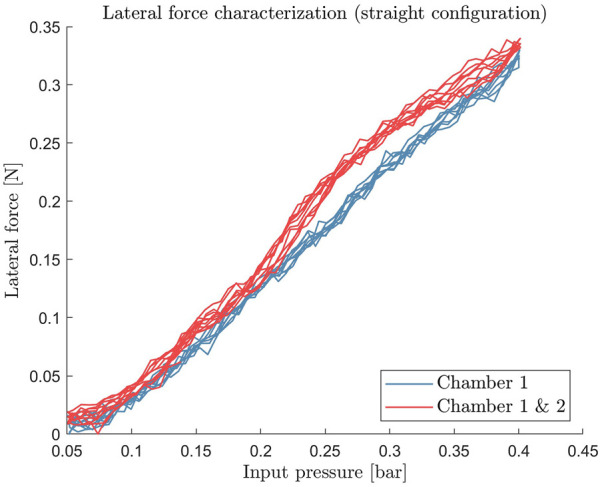
Static lateral force measurement of the soft pneumatic actuator module. The force is measured over the range of operating input pressure of 0 bar–0.4 bar.

The results of the force characterization are presented in Figure 7. The result of the force measurements shows that the module can exert a maximum lateral force of 0.33 ± 0.03 N for a single chamber and 0.34 ± 0.03 N for two-chamber activation, at 0.4 bar pressure in the reference kinematic configuration (straight). Although these force values are lower than the force required in practice, the lateral direction is the weakest direction to which the endoscope can apply force. The force values in the design requirement (Section 2.1) represent the maximum forces throughout the entire endoscopic biopsy procedure and are not restricted to a single axis. This means that these values can occur during the retraction movement during the biopsy procedure. Increasing the force that the endoscope can apply is part of our future work and is still an open problem for small-scale soft actuators.

## 5 Control strategy

### 5.1 Fitting of the characterization curve

**FIGURE 8:**
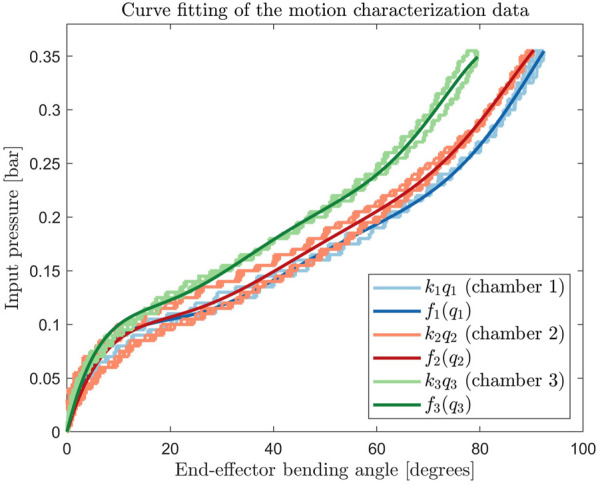
Sixth order polynomial fit of all single chamber activation motion characterization curves.

Characterization data was used to construct the forward and backward map between the input pressures *u*_*i*_ and the actuator angles *q*_*i*_. Sixth-order polynomial curves were fitted to each of the single-chamber characterization curves, as shown in Figure 8. The backward mapping from *q*_*i*_ to the input pressure *u*_*i*_ is given by:

$$u_{i}=f_{i}\left(q_{i}\right)=a_{i,6}q_{i}^{6}+a_{i,5}q_{i}^{5}+\cdots +a_{i,0},$$ (13)

where *a*_*i*,6_, *a*_*i*,5_, … , *a*_*i*,0_ are the sixth-order polynomial fitting coefficients presented in Table 2. The fitted curves were used to construct feedforward signals and adaptive feedback parameters (explained in Section 5.3).

**TABLE 2:** Polynomial fitting coefficients for the map from *q*_*i*_ to *u*_*i*_ for single-chamber characterization curves.

	*f*_1_ (*q*_1_)	*f*_2_ (*q*_2_)	*f*_3_ (*q*_3_)
*a*_6_	−1.6818 ⋅ 10^−11^	−1.7137 ⋅ 10^−11^	−4.5337 ⋅ 10^−11^
*a*_5_	5.3430 ⋅ 10^−9^	5.2358 ⋅ 10^−9^	1.1869 ⋅ 10^−8^
*a*_4_	−6.5165 ⋅ 10^−7^	−6.1841 ⋅ 10^−7^	−1.2014 ⋅ 10^−6^
*a*_3_	3.86211 ⋅ 10^−5^	3.5821 ⋅ 10^−5^	5.9594 ⋅ 10^−5^
*a*_2_	−1.1355 ⋅ 10^−3^	−1.0416 ⋅ 10^−3^	−1.4941 ⋅ 10^−3^
*a*_1_	1.6907 ⋅ 10^−2^	1.6010 ⋅ 10^−2^	2.0035 ⋅ 10^−2^
*a*_0_	0	0	0

Table 3 shows the fitting errors to the experimentally obtained characterization data. Since the hysteresis behavior was not taken into account in the fitting, this error will be compensated for by the feedback controller.

**TABLE 3:** Standard deviation of the difference between the fitted curves and the measured characterization data.

Chamber	Std. dev. of error	% of full-scale range
1	6.67 ⋅ 10^–3^ bar	1.91
2	1.08 ⋅ 10^–2^ bar	3.08
3	7.19 ⋅ 10^–3^ bar	2.05

### 5.2 Module’s workspace

Using the fitted curve and the kinematic mapping (Section 3.1), we can construct the module’s end-effector positions in 3D coordinate based on the kinematic model.

**FIGURE 9:**
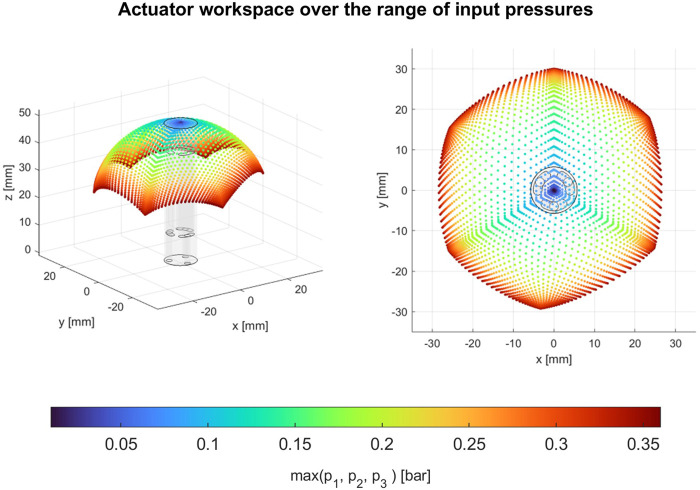
Robot’s 2D workspace in 3D space, based on the characterization data combined with module kinematics. High input pressure regions are shown in red, while low input pressure regions are shown in blue.

Figure 9 visualizes the robot workspace based on the operating input pressure level. We observe regions that require low and high input pressures to reach steady-state configuration. The maximum bending angles for the single-chamber actuation for chambers 1, 2, and 3, respectively, are 92.3°, 90.4°, and 79.5° at 0.4 bar (including 0.05 bar preloading pressure). The three other vertices in the hexagonal workspace shape (Figure 9, right image) represent the maximum bending when two chambers are pressurized simultaneously, which are 86.2°, 78.9°, and 67.9° at 0.4 bar.

### 5.3 Adaptive controller framework

The controller consists of a feedforward signal and an adaptive feedback controller. Fitted motion characterization data were used in both feedforward and feedback control. For the feedforward signal, the fitted curves were used in combination with the kinematics model (Eq. 5 and 8 to compensate for the static behavior of the module. In the case of the adaptive feedback controller, the fitted curves were used to construct the adaptive parameter used to scale the feedback gains.

We further separated the stiffness term in the system model, where it can be compensated by the feedforward term of the controller. From Eq. 9, the complete controlled system can be formulated as

$$\ddot{q}+d\left(q,\dot{q}\right)+M^{-1}K\left(q\right)q=M^{-1}\left(u_{\text{ff}}+u_{\text{fb}}\right),$$ (14)

where *u*_ff_ and *u*_fb_ are the feedforward and feedback control efforts, and

$$d\left(q,\dot{q}\right)=M^{-1}\left(C\left(q,\dot{q}\right)+D\right)\dot{q}+M^{-1}G\left(q\right)$$ (15)

contains the residual dynamics and gravity effect terms that are not known from the characterization and design parameters.

### 5.3.1 Feedforward signal using motion characterization fitting

Similar to the steps presented in Section 5.2, a map is constructed from the configuration parameters (*θ* and *ϕ*) to the required feedforward signal for each actuator *u*_ff,*i*_. Inverse kinematics was used to calculate the required actuator angles *q*_*i*_ from the reference *θ* and *ϕ*. Subsequently, the feedforward control effort is calculated using the polynomial fit *u*_*ff*_ = *f*(*q*), where $f\left(q\right)=\left[f_{1}\left(q_{1}\right),f_{2}\left(q_{2}\right),f_{3}\left(q_{3}\right)\right]T$ from Eq. 13.

The feedforward functions estimate the stiffness term *K*(*q*)*q*, which left the remaining dynamics as

$$\ddot{q}+d\left(q,\dot{q}\right)=M^{-1}u_{\text{fb}},$$ (16)

### 5.3.2 Adaptive feedback control

While feedback control can be implemented using a PID controller based on the measured orientation of the end effector, the module behavior is highly non-linear, meaning that the required controller gain might change depending on *q*_*i*_. We employ an adaptive gain-scheduling control strategy to interpolate and scale the controller gains based on the obtained characterization data. Adaptive gain scheduling as a control technique is a standard approach in the field of control theory (Bett, 2005; Anh, 2010; Åström and Wittenmark, 2013; Wu and Zheng, 2021). In this study, we introduce a technique where the characterization data of the soft actuator is used to build the gain scheduling parameter. Hence, adaptive control can be performed on the entire workspace without sampling the whole workspace itself (such as in the case when using reinforcement learning type of algorithms).

Although not all soft pneumatic actuator dynamics is known (for example, centrifugal and damping terms), the stiffness term *k*_*i*_ (*q*_*i*_) can be utilized as an adaptive scaling parameter. However, this requires the assumption that the reference trajectory dynamics is slowly varying, and therefore, the stiffness term would dominate around this low-frequency region.

This adaptive scaling parameter can be constructed by estimating *k*_*i*_ (*q*_*i*_). From Eq. 10, the estimate ${\hat{k}}_{i}\left(q_{i}\right)$ is calculated using the partial derivative of the fitted characterization curve *f*_*i*_ to *q*_*i*_:

$${\hat{k}}_{i}\left(q_{i}\right)=\frac{\partial f_{i}}{\partial q_{i}}.$$ (17)

In general, the feedback controller can be implemented in the actuator space (control effort based on *q*_*i*_) or in the configuration space (based on *θ* and *ϕ*). We opted to implement the controller in the actuator space so that the controller PID gains are set independently for each chamber. This enables a more precise tuning to counteract the non-uniformity of the module (Penning et al., 2012).

The state space form based on Eq. 16 was constructed and subsequently linearized at some chosen operating points (Bett, 2005). A PID controller was implemented and tuned at these selected points.

$$\dot{X}=AX+Bu_{\text{fb}}+\left[\begin{array}{c} 0\\ d\left(q,\dot{q}\right) \end{array}\right],$$ (18)

$$\text{where}\;A=\left[\begin{array}{cc} 0_{3\times 3} & I_{3\times 3}\\ 0_{3\times 3} & 0_{3\times 3} \end{array}\right],\;B=\left[\begin{array}{c} 0_{3\times 3}\\ M^{-1} \end{array}\right],$$

and $X=\left[q_{1},q_{2},q_{3},{\dot{q}}_{1},{\dot{q}}_{2},{\dot{q}}_{3}\right]T$ and $u_{\text{fb}}={\left[\;u_{1},u_{2},u_{3}\;\right]}^{T}$ . Eq. 18 is non-linear due to the term $d\left(q,\dot{q}\right)$ .

We define the equilibrium points $X=\bar{X}$ , and the above equation is linearized at these points, resulting in the following:

$$\Delta \dot{X}=A\Delta X+Bu_{\text{fb}}+\left[\begin{array}{c} 0\\ \bar{d}\left(t\right) \end{array}\right].$$

Thus, the feedback control effort can be formulated as

$$u_{\text{fb}}=Mu_{\text{pid}}\left(e,\dot{e}\right),$$ (19)

where *e* is the actuator space error, defined by

$$e=\bar{q}-q=\left[\begin{array}{c} {\bar{q}}_{1}-q_{1}\\ {\bar{q}}_{2}-q_{2}\\ {\bar{q}}_{3}-q_{3} \end{array}\right],$$

and *u*_pid_ contains the classical PID terms:

$$u_{\text{pid}}=K_{p}e+K_{i}\int edt+K_{d}\dot{e},$$

where *K*_*p*_, *K*_*i*_, and *K*_*d*_ are diagonal matrices containing the PID gains.

We selected two equilibrium points for each actuation chamber, one in a configuration where ${\hat{k}}_{i}$ is at minimum and the other where ${\hat{k}}_{i}$ is at maximum. The actuator space configuration when the adaptive parameter ${\hat{k}}_{i}$ is at minimum is defined as ${\bar{q}}_{i}^{\;min}$ , and similarly the actuator space configuration at maximum ${\hat{k}}_{i}$ is defined as ${\bar{q}}_{i}^{\;max}$ . In this case, the two equilibrium points are ${\bar{q}}_{i}^{\;min}=20{}^{\circ},20{}^{\circ},17{}^{\circ}$ and ${\bar{q}}_{i}^{\;max}=92{}^{\circ},90{}^{\circ},73{}^{\circ}$ respectively for *i* = 1, 2, 3. Using these equilibrium points, the gains for each actuation chamber (e.g., *K*_*p*,*i*_) were interpolated (Bett, 2005) for the complete bending range using:

$$K_{p,i}=\frac{\left({\hat{k}}_{i}\left(q_{i}\right)-{\hat{k}}_{i}\left({\bar{q}}_{i}^{\;min}\right)\right)}{{\hat{k}}_{i}\left({\bar{q}}_{i}^{\;max}\right)-{\hat{k}}_{i}\left({\bar{q}}_{i}^{\;min}\right)}{\bar{K}}_{p,i}^{\;min}+\frac{\left({\hat{k}}_{i}\left({\bar{q}}_{i}^{\;max}\right)-{\hat{k}}_{i}\left(q_{i}\right)\right)}{{\hat{k}}_{i}\left({\bar{q}}_{i}^{\;max}\right)-{\hat{k}}_{i}\left({\bar{q}}_{i}^{\;min}\right)}{\bar{K}}_{p,i}^{\;max}$$ (20)

where ${\bar{K}}_{p,i}^{\;min}$ and ${\bar{K}}_{p,i}^{\;max}$ are the tuned P gains at the two respective equilibrium points.

Similarly, this adaptive gain tuning method was also performed for the I and D gains. We tuned the PID gains at the two equilibrium configurations ${\bar{q}}_{i}^{\;min}$ and ${\bar{q}}_{i}^{\;max}$ , and interpolated the gains for the entire operating range using Eq. 20.

### 5.4 Experimental validation

A trajectory tracking experiment was conducted to validate the performance of the control method. Using the same setup as the experimental characterization experiment (Section 2.4, the bending angles were measured at the tip of the endoscope using an electromagnetic tracker. Subsequently, the feedback and feedforward control efforts are calculated using the methods described above.

A square reference trajectory was used to validate the tracking accuracy of the soft pneumatic system. The maximum bending angles are 35° (at the corners of the square trajectory), and the endoscope tip traced the entire square with a period of 30 s. The tracking experiment is performed over 2 laps of the square trajectory.

## 6 Results and discussions

### 6.1 Tracking accuracy results

**FIGURE 10:**
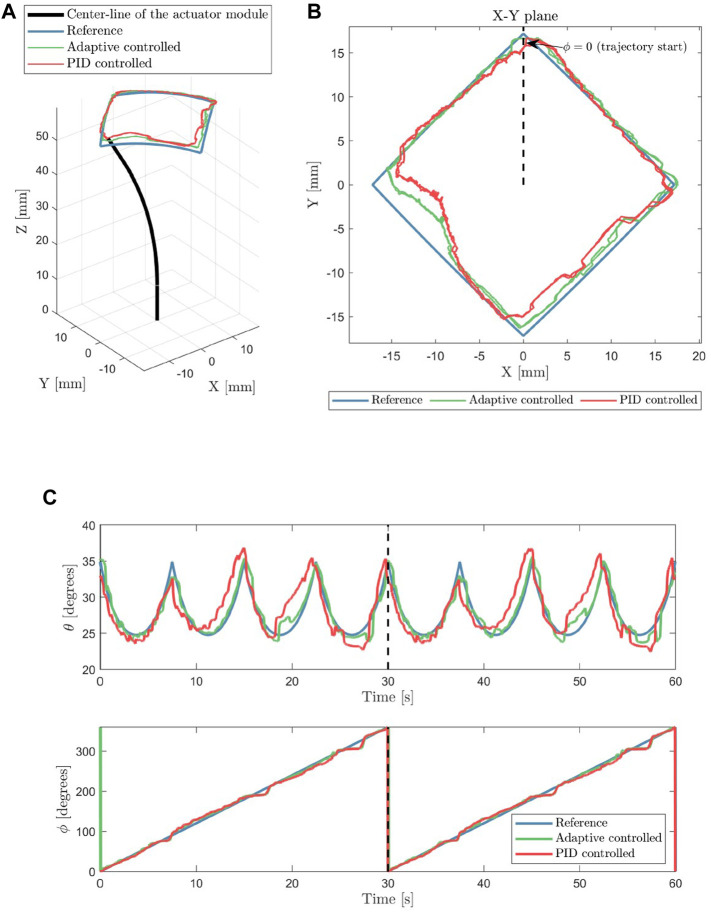
The visualization of the actuator tracking a square reference trajectory in 3D is presented in **(A)**, while the tip position from the top-down view is shown in **(B)**. The tracking results of the tip orientation angles (*θ* and *ϕ*) are shown in **(C)**. The black dashed lines indicate the start of the second lap of the square trajectory.

The result of the tracking experiment following the trajectory is shown in Figure 10. Keep in mind that the controller controls the tip angles, not the position in Cartesian coordinates, as motivated in Section 3. The measured tip positions shown in Figures 10A, B, are calculated using the kinematic model described in Section 3.1.

Over 2 laps of the square reference trajectory, the actuator was able to track the trajectory with a mean absolute error of 0.75° for the bending angle *θ* and 1.8° for the phase angle *ϕ*. The mean absolute error for the bending angle *θ* is 2.1% of the reference trajectory’s bending range and 0.5% for the phase angle *ϕ*. Using the proposed adaptive controller framework, the endoscope was able to track the reference trajectory angles with reasonable accuracy.

### 6.2 Discussions

**FIGURE 11:**
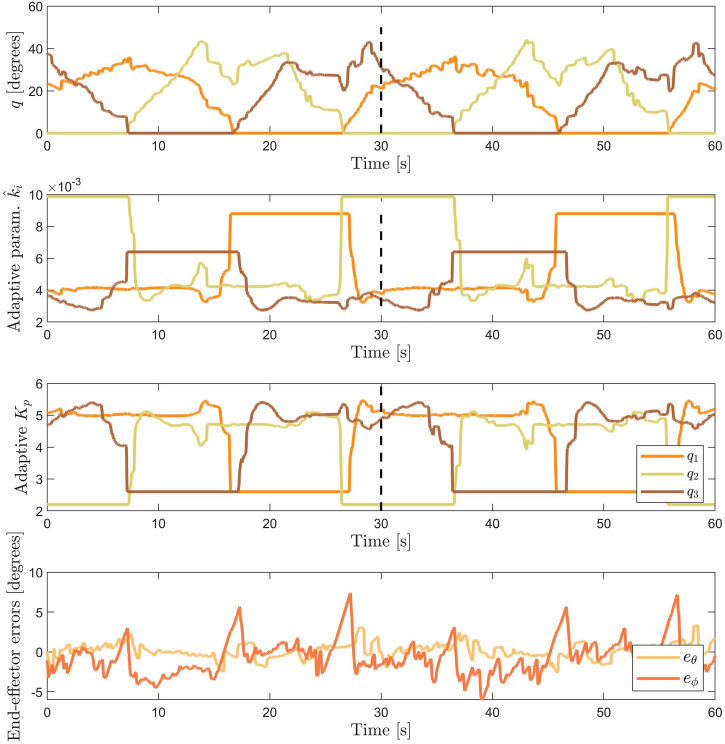
From top to bottom: the actuator space bending angles *q*, the adaptive parameter ${\hat{k}}_{i}$ , the adaptive *K*_*p*_ gain, and the tracking error of the adaptive controller over the length of the square reference trajectory. The tuning for the maximum and minimum adaptive parameters are ${\bar{K}}_{p}^{\;max}=5.4,5.1,5.4$ and ${\bar{K}}_{p}^{\;min}=2.6,2.2,2.6$ respectively for chamber 1, 2, and 3.

The results of the adaptive controller indicate closer tracking of the reference trajectory compared to conventional PID control, as shown in Figures 10A–C. The adaptive parameter based on the stiffness of the characterized module enables the adaptive PID controller to be tuned with a higher gain at bent configuration (Figure 11). In contrast, the conventional PID controller can only be tuned at lower constant *K*_*p*_ gain, before it exhibits unstable oscillations. The adaptive controller is able to set its gain to a higher value in the bent configuration and lower in the straight configuration, while the conventional PID controller is fixed at constant gain values.

The error at the left corner in Figure 10B are present for both the adaptive and conventional PID controlled actuator. At this location, the tip orientation is highly dependent on the contribution of chamber 2. Initially, we think that this error is caused by the uncompensated hysteresis behavior (which is the largest in chamber 2). However, if we compare Figures 10B, C, this error is more apparent in the position plot (Figure 10B) than the orientation plot (Figure 10C, visible at *t* ≈ 20 sec and *t* ≈ 50 sec). This indicates that the actuator is able to point to the reference direction, however, the resulting tip position is not correct. Therefore, it means that PCC kinematic assumption does not perform well specifically for this configuration. This may have been caused by the uneven thickness of the inner silicone wall due to manual fabrication, which caused the actuator to bend in a different amount of curvature over the length of the actuator. The PCC assumption is a major source of error for the end-effector position estimate. Moreover, using PCC introduces a kinematic singularity in the straight configuration. Alternative solutions, such as variable curvature kinematics in Huang et al. (2021), might solve the aforementioned problems.

Our approach in using data-driven adaptive control based on characterization is a way to compensate for real-world effects that are difficult to model, such as material non-linearity and non-uniformity due to design and fabrication steps. Although the non-linearity in this case includes the change in sensitivity over different bending configurations, the compensation for hysteresis and other path-dependent behaviors has not been compensated yet. The hysteresis behavior might cause limit-cycle behavior in the resulting controlled behavior. This is shown as the low magnitude but high-frequency motion in the tracking results in Figure 10.

The dynamics of the pressure regulator significantly affects the tracking results. The resolution of the pressure regulators was limited to 8 bits over 2 bar full-scale range. This limitation can be noticed in Figures 10B, C where the measured tip orientation showed a staircase-like behavior, where the step size of the staircase indicates the resolution of the pneumatic regulator hardware. This behavior sets the lower bound of the tracking error in our implementation.

The initial setting of the outer reinforcement fiber during the fabrication of the SPA strongly influences the maximum bending angle of the module (as presented in the Supplementary Material S1). Compressing the reinforcement fiber during fabrication will increase the maximum bending response limit of the soft actuator. However, this increases the risk of non-uniform bending response magnitude between individual actuation chambers due to uneven compression due to the manual fabrication process. Consequently, this phenomenon also leads to non-uniform separation angles *ϕ*_sep_ between the actuation chambers, namely, *ϕ*_sep_ between chambers 1 and 2, 2 and 3, 1 and 3.

Since the scope of this study does not include the overall endoscope design, the response of the endoscopic actuator when a camera or tools are included might add non-linearity components to the actuator response. We expect that the complex interaction from the added tool/tube will increase the amount of non-linearity in the function map. More tuning points could be used to tune the adaptive controller to compensate for the complex actuator behavior.

We realize that further investigation is needed related to the response of the controller against external perturbations. In this work, we focused on the accuracy and repeatability of the soft pneumatic actuator, especially with regard to how the controller can compensate for the complex and non-uniform behavior of the actuator. The response of the soft pneumatic endoscope to external perturbations will be addressed in our future work. Furthermore, the actuator response should be investigated for different external impulse directions in different configurations.

## 7 Conclusion

We presented the FE simulation, characterization, and control of a fiber reinforced soft pneumatic actuator. The module was characterized to determine the mapping between the required pressures in the actuation chambers and the orientation angles of the tip. These experimentally obtained characterization data were used to construct an adaptive controller to follow a prescribed tip orientation trajectory in 2D. Using the data-driven adaptive control framework, the non-linear material behavior and non-uniformity of the fiber reinforced SPA can be compensated while maintaining the ability to intuitively tune and adjust the behavior of the controller. The results of the tracking experiment show that the proposed control method can follow a prescribed reference trajectory in real time. Our future work will focus on improving the accuracy of the kinematic model and including a hysteresis model in actuator space mapping, to further improve the control of a fiber reinforced soft pneumatic actuator.

## Data Availability

The raw data supporting the conclusion of this article will be made available by the authors, without undue reservation.
