# Supplementary figures and images for: Adaptive control of a soft pneumatic actuator using experimental characterization data
Source: Front Robot AI. 2023 Mar 15;10:1056118. doi: 10.3389/frobt.2023.1056118 (PMC10050439; doi:10.3389/frobt.2023.1056118)

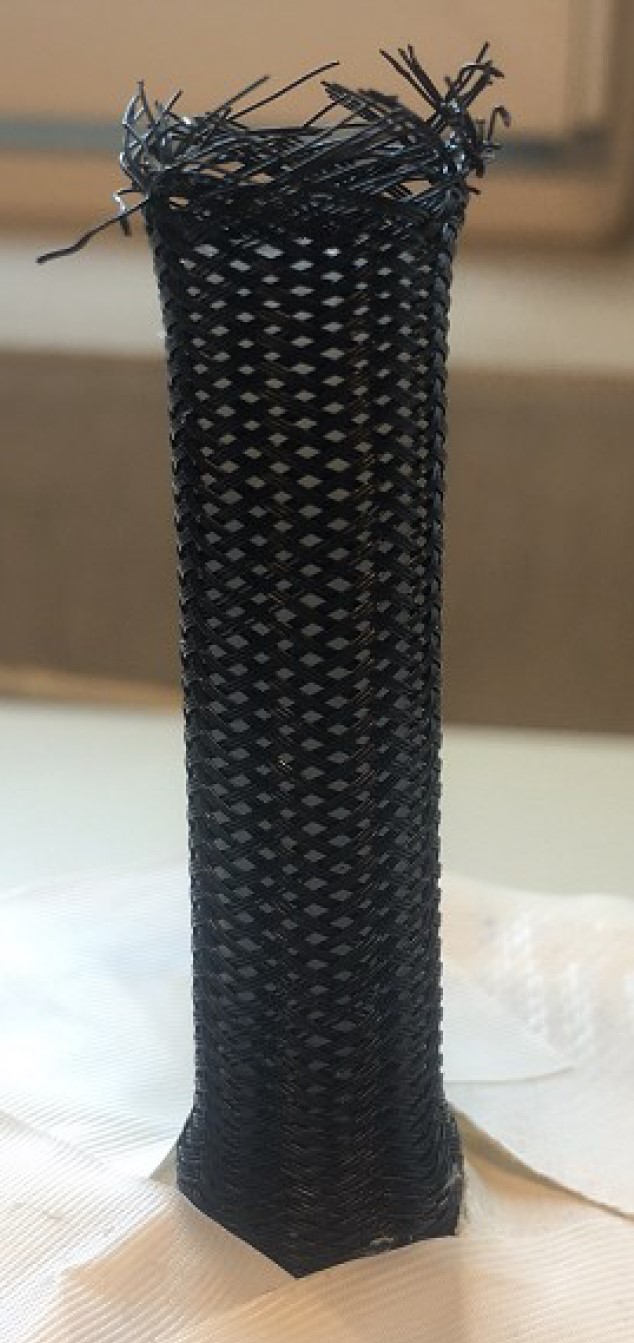

Supplement: Supplementary file 2 [file Image3.JPEG]

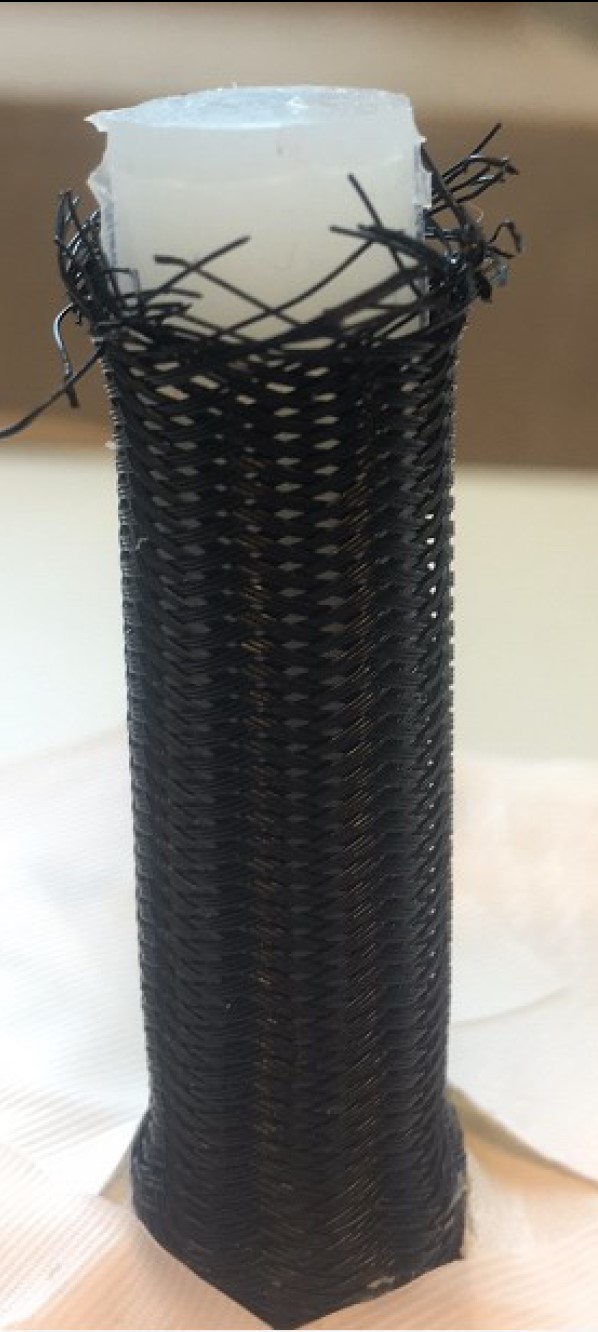

Supplement: Supplementary file 4 [file Image1.JPEG]

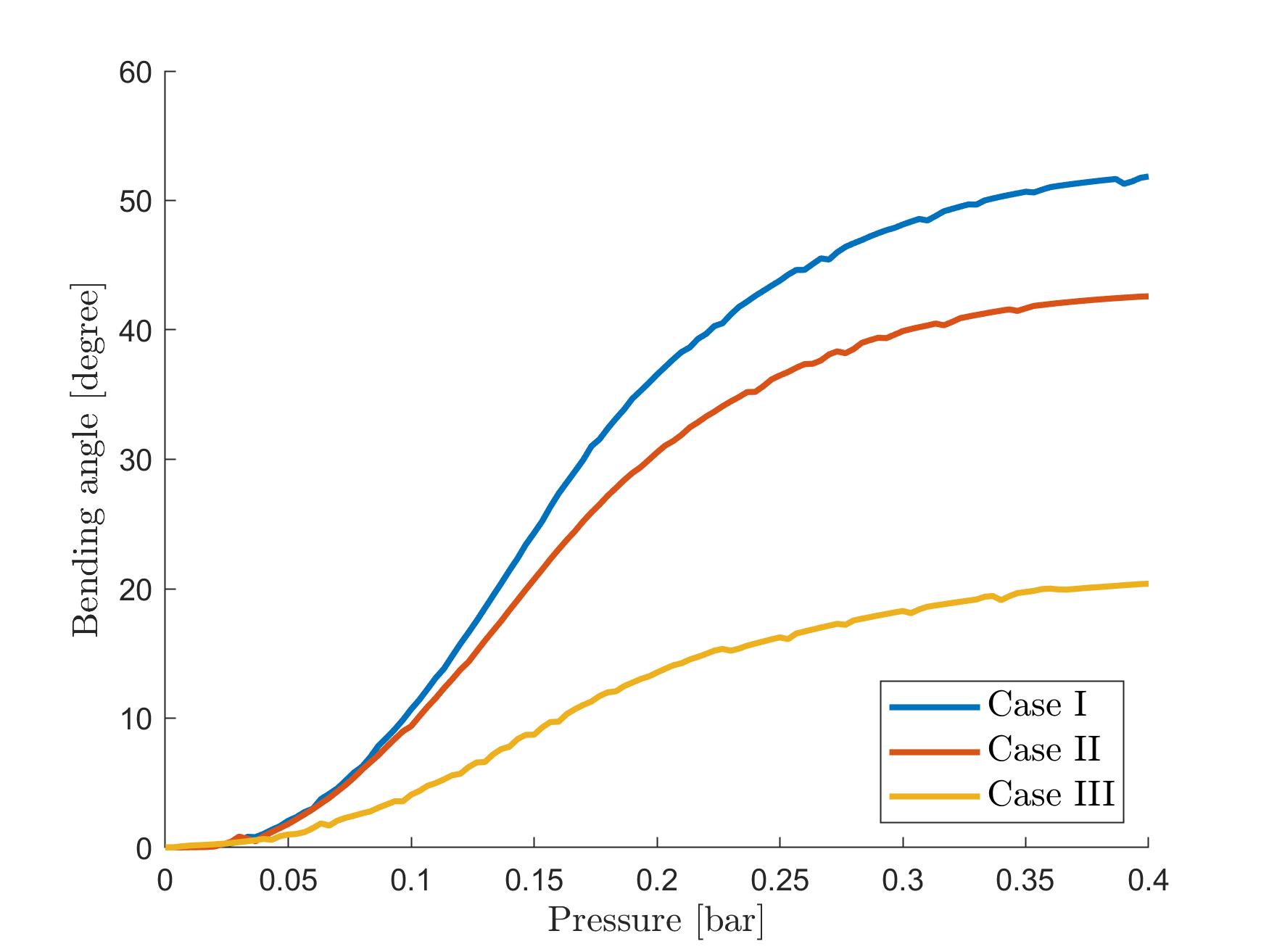

Supplement: Supplementary file 5 [file Image4.JPEG]

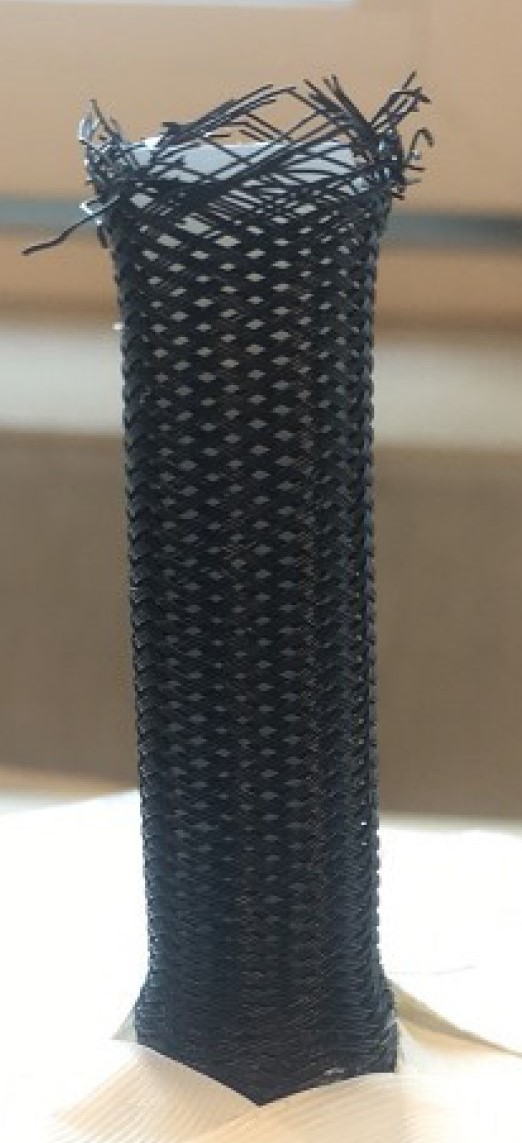

Supplement: Supplementary file 6 [file Image2.JPEG]
