# Supplementary material for: Adaptive control of a soft pneumatic actuator using experimental characterization data
Source: Front Robot AI. 2023 Mar 15;10:1056118. doi: 10.3389/frobt.2023.1056118 (PMC10050439; doi:10.3389/frobt.2023.1056118)
Supplement: Supplementary file 3 [file Presentation1.pdf]

Supplementary Material – Adaptive Control of a Soft Pneumatic Actuator Using Experimental Characterization Data

Yoeko Xavier Mak [1,*], Hamid Naghibi [1], Yuanxiang Lin [1] and Momen Abayazid [1]

[1] *Robotics and Mechatronics group, Faculty of Electrical Engineering, Mathematics and Computer Science, Technical Medical (TechMed) Centre, University of Twente, 7500 AE Enschede, the Netherlands*

Correspondence*:
Momen Abayazid
m.abayazid@utwente.nl

1 EFFECT OF OUTER FIBER VARIATION TO THE BENDING RESPONSE

We have observed that the manner in which the reinforcement fiber is placed on the exterior of the inner module during the fabrication process greatly influences the resulting bending response of the soft pneumatic actuator (SPA). In particular, how much the reinforcement fiber is compressed when it is fixed to the tip of the inner module sets the maximum bending limit of the actuator. To show how much this factor influences the bending response, we performed an additional experiment in which we performed bending motion characterization for three different fiber placement settings.

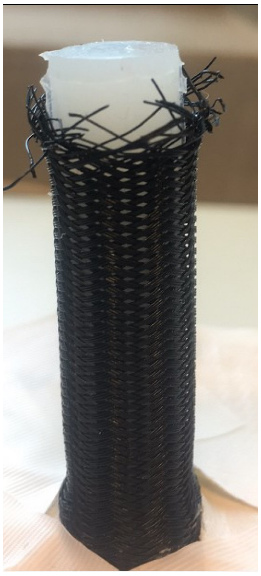

Figure 1a. Case I

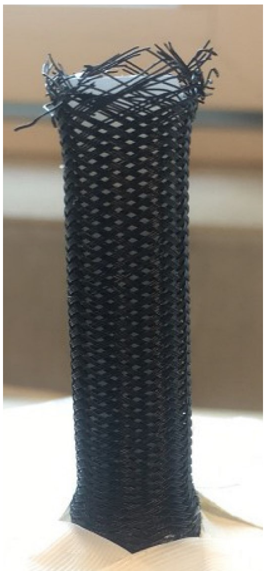

Figure 1b. Case II

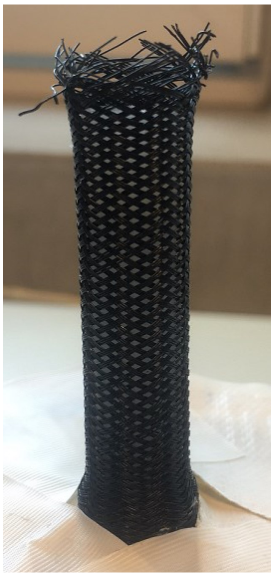

Figure 1c. Case III

Figure 1. The outer reinforcement fiber is cut to the same length (in its original pre-fitted diameter) and set to the exterior of the inner module in three different settings.

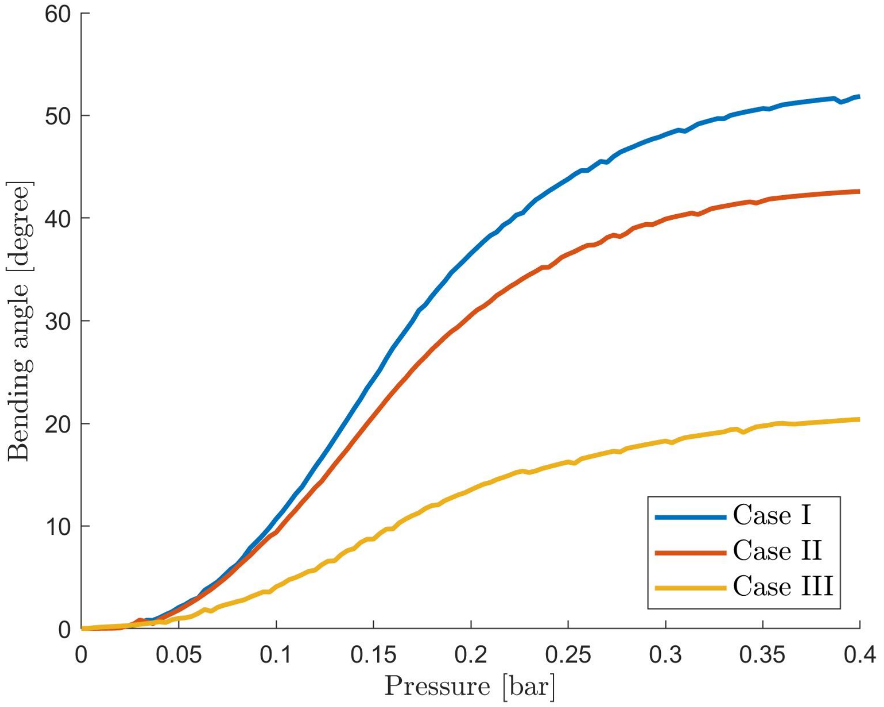

Figure 2. Maximum bending angle response for three different fiber reinforcement placement settings.

The three different settings are tested, as shown in Fig. 1. In case I, the fibers are compressed as much as possible, and the end is fixed to the cap section of the inner module. In case II, the fibers are slightly elongated, and the fibers end are fixed to the tip of the module. In case III, the outer fibers are stretched to more than the length of the silicone inner module.

The result of this experiment is presented in Fig. 2. The bending angle at the maximum operating pressure limit of 0.4 bar is the highest for Case I. Therefore, compressing the reinforcement fiber before gluing the ends to the inner module will help to achieve a wider bending range.

The reason for this is that the braiding pattern of the reinforcement fiber sets the saturation behavior for the actuator's extension mode. When the fiber braiding is mostly horizontal (perpendicular to the actuator length), as in Case I, the reinforcement fiber structure has the least resistance to extension movement. On the other hand, in Case III, where the fiber braiding is closer to its lengthening saturation limit, the SPA must overcome this tension from the reinforcement fiber structure, which lowers its maximum bending response.

However, uneven compression during the fabrication process will lead to a nonuniform maximum bending limit between the three actuation chambers. Consequently, this will also create a nonuniform separation angle ϕ_{sep} between the bending planes of q_1, q_2, and q_3, which must be taken into account in the robot kinematics (Section 3.1).
